# Supplementary figures and images for: Optimal timing of a colonoscopy screening schedule depends on adenoma detection, adenoma risk, adherence to screening and the screening objective: A microsimulation study
Source: PLoS One. 2024 May 24;19(5):e0304374. doi: 10.1371/journal.pone.0304374 (PMC11125540; doi:10.1371/journal.pone.0304374)

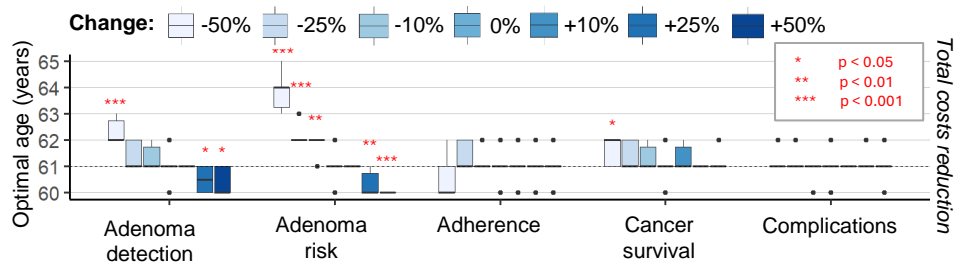

Supplement: S1 Fig — Selected model parameters were varied by up to ±50%. Screening time points for a one-colonoscopy program are indicated for optimal CRC reduction of total costs. Dashed lines indicate the optimal schedule without parameter changes. Significance is tested using the Wilcoxon test against a distribution without parameter changes. *p < 0.05, **p < 0.01. ***p < 0.001. For all statistics N = 10 simulations were used with a population of 20 million individuals. (PDF) [file pone.0304374.s001.pdf]

**A**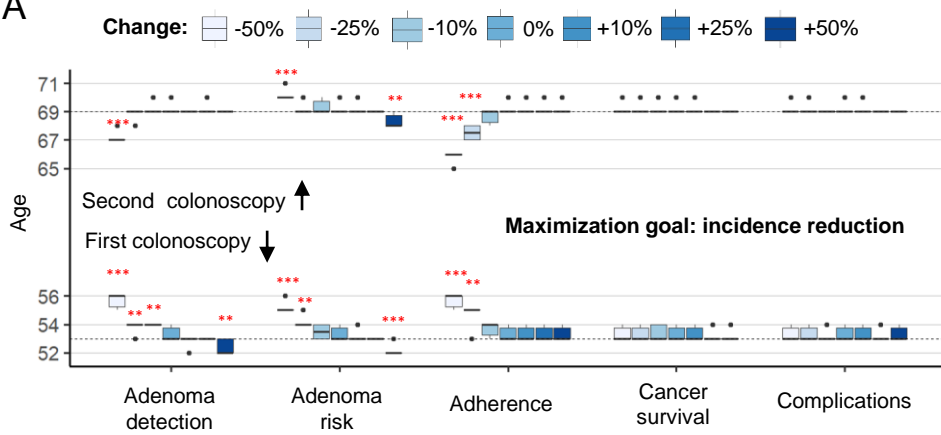**B**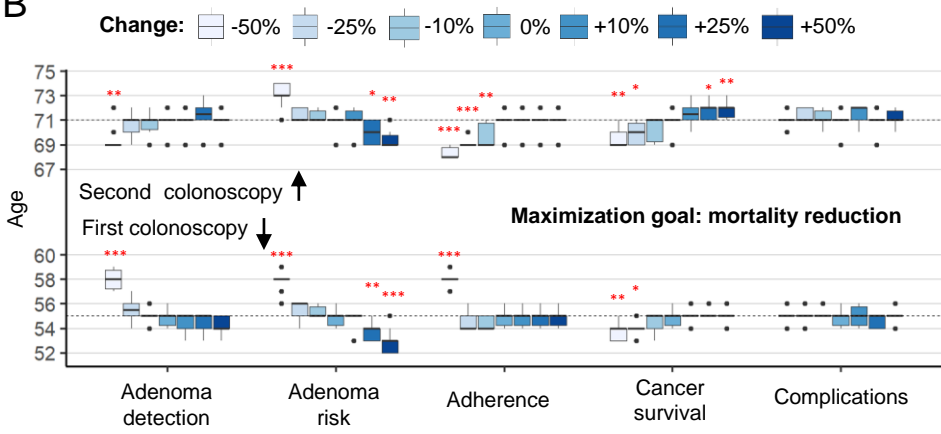**C**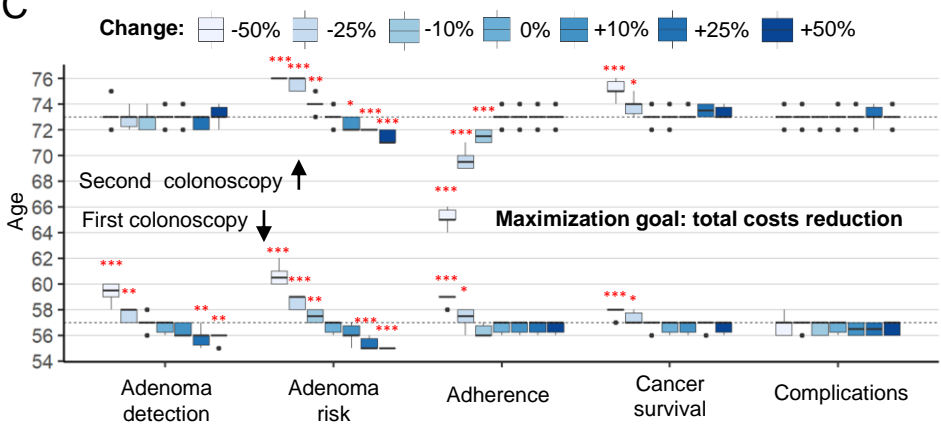

Supplement: S2 Fig — As Fig 1B, only optimal time points for a two-colonoscopy program are indicated with optimal CRC reduction of total costs. (PDF) [file pone.0304374.s002.pdf]

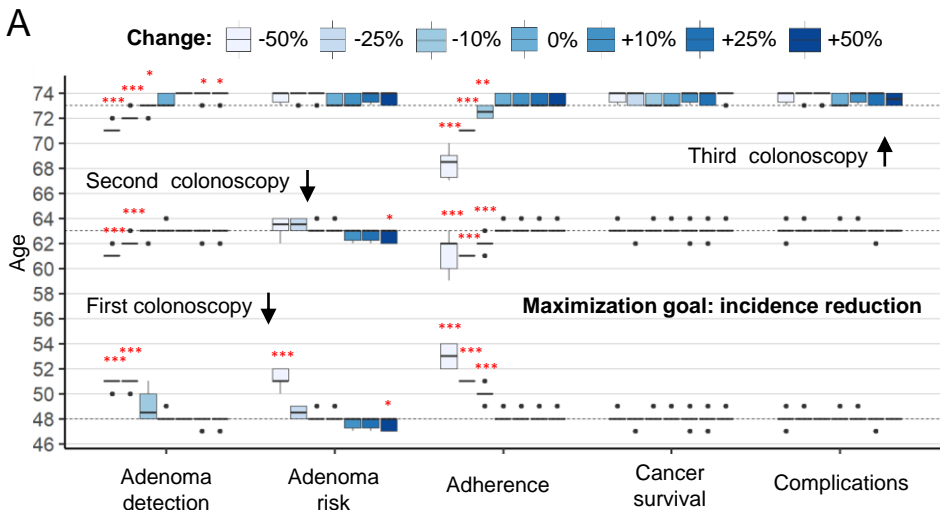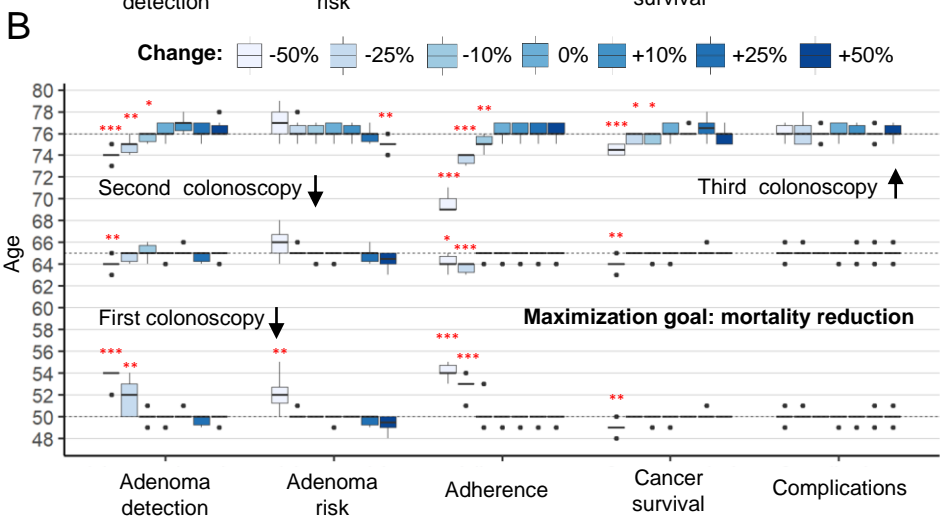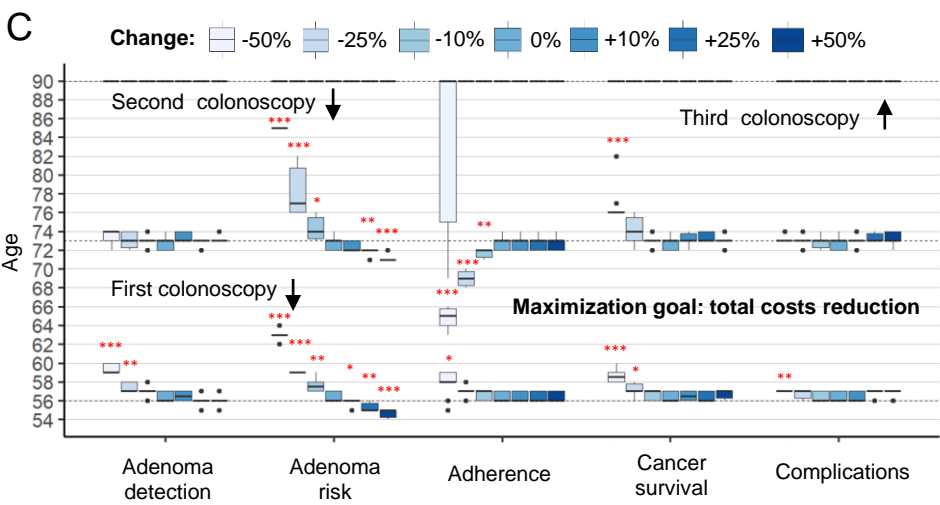

Supplement: S3 Fig — As Fig 1B, only optimal time points for a three-colonoscopy program are indicated with optimal CRC reduction of total costs. (PDF) [file pone.0304374.s003.pdf]

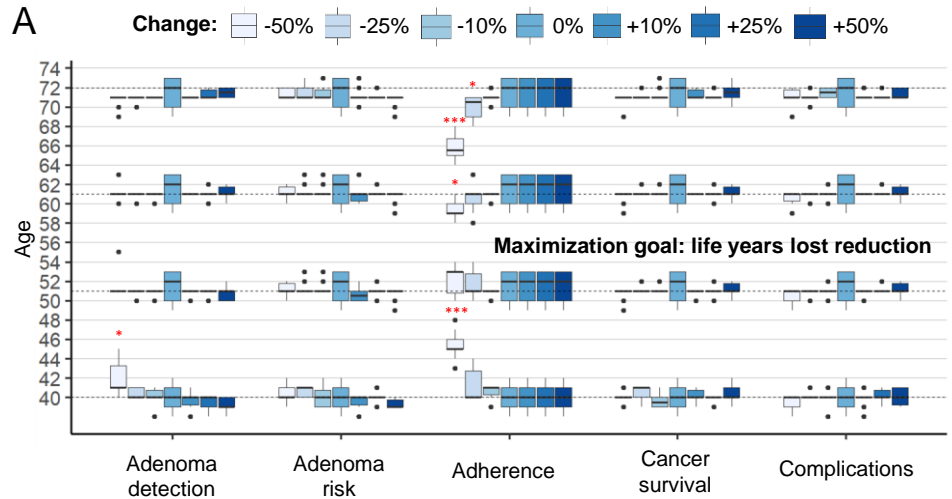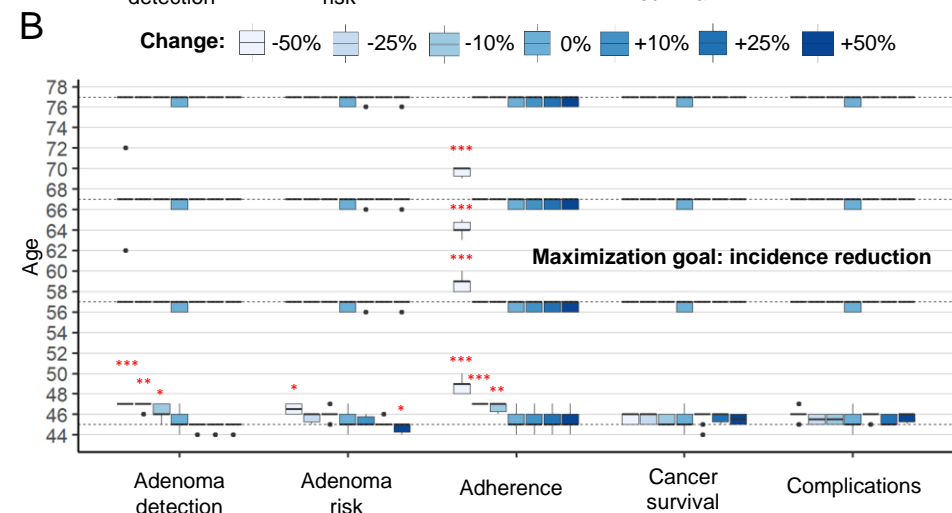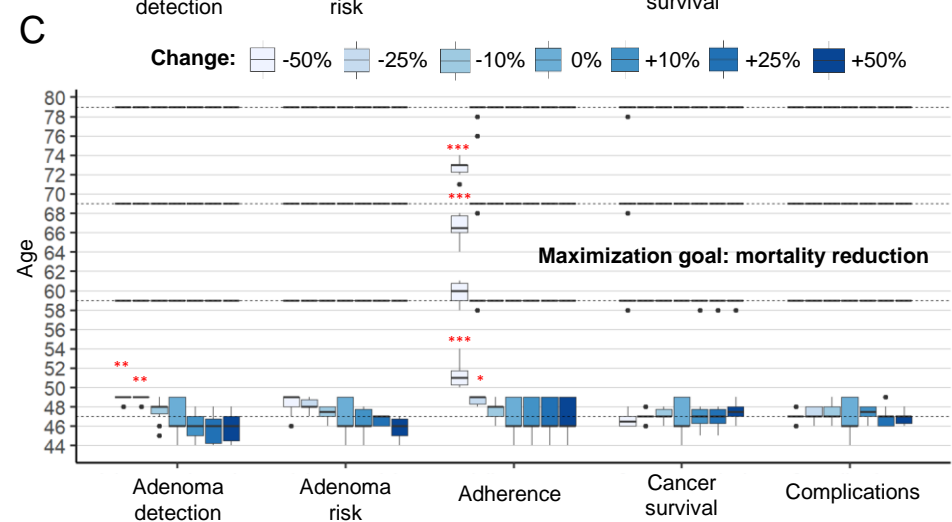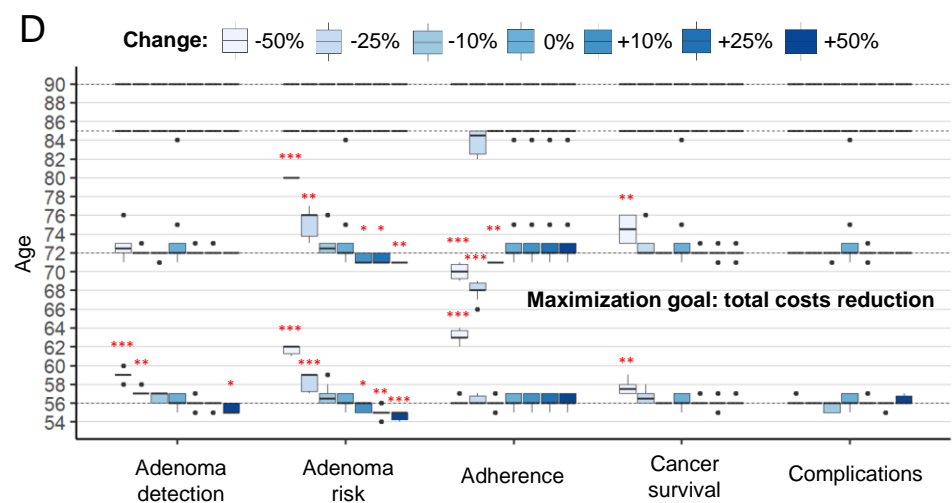

Supplement: S4 Fig — As Fig 1B, only optimal time points for a four-colonoscopy program are indicated with optimal CRC reduction of total costs. (PDF) [file pone.0304374.s004.pdf]
