# Supplementary material for: Optimal timing of a colonoscopy screening schedule depends on adenoma detection, adenoma risk, adherence to screening and the screening objective: A microsimulation study
Source: PLoS One. 2024 May 24;19(5):e0304374. doi: 10.1371/journal.pone.0304374 (PMC11125540; doi:10.1371/journal.pone.0304374)
Supplement: S1 Table — (DOCX) [file pone.0304374.s005.docx]

Additional file 1: Table S1: Benchmarks and results for CMOST calibration^1^

**Benchmark 1:** Early adenoma prevalence (percent of population with ≥1 adenoma) ^2-3^

| Age (years) | 25 | | 35 | | 45 | | 60 | | 70 | | 80 | |
| --- | --- | --- | --- | --- | --- | --- | --- | --- | --- | --- | --- | --- |
|  | Benchmark | CMOST | Benchmark | CMOST | Benchmark | CMOST | Benchmark | CMOST | Benchmark | CMOST | Benchmark | CMOST |
| Early adenomas, overall | 4.5% | 2.6% | 7.2% | 6% | 9.4% | 11.9% | 27% | 25.7% | 39.5% | 33.9% | 42.6% | 38.5% |
| Early adenomas, male | 5.7% | 3.6% | 9.1% | 8.1% | 11.9% | 15.6% | 34.1% | 32.5% | 49.9% | 41.7% | 53.7% | 46.6% |
| Early adenoma, female | 3.3% | 1.6% | 5.4% | 4% | 7% | 8.4% | 20% | 19.3% | 29.2% | 27.1% | 31.5% | 32.3% |

**Benchmark 2:** Advanced adenoma prevalence (percent of population with ≥1 advanced adenoma) ^2, 4^

| Age (years) | 57 | | 62 | | 67 | | 72 | | 77 | | 85 | |
| --- | --- | --- | --- | --- | --- | --- | --- | --- | --- | --- | --- | --- |
|  | Benchmark | CMOST | Benchmark | CMOST | Benchmark | CMOST | Benchmark | CMOST | Benchmark | CMOST | Benchmark | CMOST |
| Advanced adenomas, overall | 4.8% | 4.3% | 5.8% | 5.6% | 6.6% | 6.8% | 7.7% | 7.9% | 8.1% | 8.5% | 8.4% | 8.9% |
| Advanced adenomas, male | 6.2% | 5.5% | 7,5% | 7.1% | 8.4% | 8.7% | 9.4% | 10% | 9.7% | 11% | 9.5% | 11.8% |
| Advanced adenoma, female | 3.4% | 3.2% | 4.2% | 4.1% | 4.8% | 5.1% | 5.8% | 6% | 6.5% | 6.5% | 7.3% | 7% |

**Benchmark 3:** Cancer incidence (per 100,000 per year) based on SEER 1988-2002 data ^5^

| Age (years) | Incidence overall | | Incidence male | | Incidence female | |
| --- | --- | --- | --- | --- | --- | --- |
|  | Benchmark | CMOST | Benchmark | CMOST | Benchmark | CMOST |
| 22 | 1 | 1 | 1 | 1 | 0 | 1 |
| 27 | 1 | 2 | 1 | 2 | 2 | 2 |
| 32 | 3 | 3 | 3 | 4 | 3 | 2 |
| 37 | 7 | 6 | 7 | 9 | 6 | 4 |
| 42 | 13 | 12 | 14 | 13 | 12 | 11 |
| 47 | 26 | 32 | 28 | 39 | 23 | 26 |
| 52 | 50 | 47 | 56 | 60 | 43 | 35 |
| 57 | 86 | 84 | 102 | 106 | 71 | 65 |
| 62 | 138 | 141 | 166 | 167 | 110 | 118 |
| 67 | 207 | 226 | 252 | 273 | 163 | 185 |
| 72 | 281 | 282 | 336 | 338 | 227 | 234 |
| 77 | 366 | 384 | 434 | 458 | 299 | 326 |
| 82 | 456 | 443 | 531 | 529 | 381 | 382 |
| 87 | 454 | 504 | 495 | 614 | 413 | 436 |

**Benchmark 4:** Adenoma stage distribution ^6-7^

| Adenoma stage | I | | II | | III | | IV | | V | | VI | |
| --- | --- | --- | --- | --- | --- | --- | --- | --- | --- | --- | --- | --- |
|  | Benchmark | CMOST | Benchmark | CMOST | Benchmark | CMOST | Benchmark | CMOST | Benchmark | CMOST | Benchmark | CMOST |
| Percentage of all early adenomas (%) | 38.4 | 46.4 | 29.7 | 26.2 | 23.1 | 19.9 | 8.7 | 7.5 |  |  |  |  |
| Percentage of all advanced adenomas (%) |  |  |  |  |  |  |  |  | 79.8 | 79 | 20.2 | 21 |

**Benchmark 5:** Distribution of simultaneous multiple adenomas in a population of 54-74 year old individuals ^8^

|  | Benchmark | CMOST | Benchmark | CMOST | Benchmark | CMOST | Benchmark | CMOST | Benchmark | CMOST |
| --- | --- | --- | --- | --- | --- | --- | --- | --- | --- | --- |
| Number of adenomas | 1 | | 2 | | 3 | | 4 | | ≥5 | |
| Percentage of individuals with n adenomas | 36 | 29 | 16 | 12 | 5 | 6 | 4 | 4 | 3 | 3 |

**Benchmark 6:** Relative likelihood of an adenoma transforming to carcinoma ^7, 9^

| Adenoma stage | I | | | II | | III | | IV | | V | | | VI | |
| --- | --- | --- | --- | --- | --- | --- | --- | --- | --- | --- | --- | --- | --- | --- |
|  | Benchmark | CMOST | | Benchmark | CMOST | Benchmark | CMOST | Benchmark | CMOST | Benchmark | CMOST | | Benchmark | CMOST |
| Relative likelihood of transformation | 0.002 | | 0.05 | 0.21 | 0.07 | 0.3 | 0.3 | 1.06 | 0.33 | 12.8 | | 9.5 | 85.5 | 89.7 |

**Benchmark 7:** Percentage of rectal carcinoma of all carcinoma ^5^

| Rectal carcinoma percentage (age group) | 61-65 years | | 71-75 years | |
| --- | --- | --- | --- | --- |
|  | Benchmark | CMOST | Benchmark | CMOST |
| Percentage overall | 31.2 | 29.1 | 25.8 | 27.6 |
| Percentage male | 34.1 | 32.8 | 28.6 | 29.3 |
| Percentage female | 28.3 | 25 | 23.0 | 25.8 |

**Benchmark 8:** Stage distribution of symptomatic cancer (i.e. cancer detected due to symptoms) ^10^

| Cancer stage | Stage I | | Stage II | | Stage III | | Stage IV | |
| --- | --- | --- | --- | --- | --- | --- | --- | --- |
|  | Benchmark | CMOST | Benchmark | CMOST | Benchmark | CMOST | Benchmark | CMOST |
| Percentage of all cancer | 18.9 | 21.7 | 27.7 | 32.9 | 29.9 | 25.5 | 23.5 | 19.8 |

**Benchmark 9:** Stage distribution of asymptomatic cancer (i.e. cancer detected during screening) ^10-11^

|  | Benchmark | CMOST | Benchmark | CMOST | Benchmark | CMOST | Benchmark | CMOST |
| --- | --- | --- | --- | --- | --- | --- | --- | --- |
| Cancer stage | Stage I | | Stage II | | Stage III | | Stage IV | |
| Percentage of all cancer | 39.5 | 46.9 | 34.7 | 31.5 | 17.3 | 14.9 | 8.5 | 6.6 |

References

1. Meher K. Prakash, B. M., CMOST: an open-source framework for the microsimulation of colorectal cancer screening strategies. **2017,** *17*.

2. Jemal, A.; Siegel, R.; Ward, E.; Hao, Y.; Xu, J.; Thun, M. J., Cancer statistics, 2009. *CA A Cancer J Clin* **2009,** *59* (4), 225-249.

3. Center, M. M.; Jemal, A.; Ward, E., International trends in colorectal cancer incidence rates. *Cancer Epidemiology Biomarkers & Prevention* **2009,** *18* (6), 1688-1694.

4. Jones, S.; Chen, W.-d.; Parmigiani, G.; Diehl, F.; Beerenwinkel, N.; Antal, T.; Traulsen, A.; Nowak, M. A.; Siegel, C.; Velculescu, V. E., Comparative lesion sequencing provides insights into tumor evolution. *Proceedings of the National Academy of Sciences* **2008,** *105* (11), 4283-4288.

5. Winawer, S.; Fletcher, R.; Rex, D.; Bond, J.; Burt, R.; Ferrucci, J.; Ganiats, T.; Levin, T.; Woolf, S.; Johnson, D., Colorectal cancer screening and surveillance: clinical guidelines and rationale—update based on new evidence. *Gastroenterology* **2003,** *124* (2), 544-560.

6. Heitman, S. J.; Ronksley, P. E.; Hilsden, R. J.; Manns, B. J.; Rostom, A.; Hemmelgarn, B. R., Prevalence of Adenomas and Colorectal Cancer in Average Risk Individuals: A Systematic Review and Meta-analysis. *Clinical Gastroenterology and Hepatology* **2009,** *7* (12), 1272-1278.

7. Lieberman, D. A.; Rex, D. K.; Winawer, S. J.; Giardiello, F. M.; Johnson, D. A.; Levin, T. R., Guidelines for Colonoscopy Surveillance After Screening and Polypectomy: A Consensus Update by the US Multi-Society Task Force on Colorectal Cancer. *Gastroenterology* **2012,** *143* (3), 844-857.

8. Williams, A. R.; Balasooriya, B. A.; Day, D. W., Polyps and cancer of the large bowel: a necropsy study in Liverpool. *Gut* **1982,** *23* (10), 835-42.

9. Kuntz, K. M.; Lansdorp-Vogelaar, I.; Rutter, C. M.; Knudsen, A. B.; Van Ballegooijen, M.; Savarino, J. E.; Feuer, E. J.; Zauber, A. G., A Systematic Comparison of Microsimulation Models of Colorectal Cancer: The Role of Assumptions about Adenoma Progression. *Med Decis Making* **2011,** *31* (4), 530-539.

10. Heitman, S. J.; Hilsden, R. J.; Au, F.; Dowden, S.; Manns, B. J., Colorectal cancer screening for average-risk North Americans: an economic evaluation. *PLoS Med* **2010,** *7* (11), e1000370.

11. Schoepfer, A.; Marbet, U. A., Colonoscopic findings of symptomatic patients aged 50 to 80 years suggest that work-up of tumour suspicious symptoms hardly reduces cancer-induced mortality. *Swiss medical weekly* **2005,** *135* (45-46), 679-83.
