# Supplementary material for: Optimal timing of a colonoscopy screening schedule depends on adenoma detection, adenoma risk, adherence to screening and the screening objective: A microsimulation study
Source: PLoS One. 2024 May 24;19(5):e0304374. doi: 10.1371/journal.pone.0304374 (PMC11125540; doi:10.1371/journal.pone.0304374)
Supplement: S2 Table — (DOCX) [file pone.0304374.s006.docx]

Additional file 2: Table S2: Basic parameters for CMOST simulations

| Population | 20 million |
| --- | --- |
| Simulation years | 0..100 |
| Adherences | 100% for screening and surveillance colonoscopies |
| Adenoma detection rate during colonoscopy | Adenoma detection by stage from 1 to 6: 65%, 75%, 81%, 87%, 95%, 95%  Cancer detection rate by stage from I to IV: 95%, 95%, 95%, 100% |
| Colonoscopy cost, USD | Colonoscopy without lesions: 916.66  Colonoscopy with neoplasms: 1205.07 |
| Cancer treatment costs, USD: | In the first year after diagnosis:  Stage I: 44658  Stage II: 59938  Stage III: 72751  Stage IV: 94702  In years 2-4 after diagnosis, per year:  Stage I: 3713  Stage II: 3494  Stage III: 4895  Stage IV: 14826  In the last year before death due to CRC:  Stage I: 77682  Stage II:77373  Stage III:81616  Stage IV:107580  In the last year of life in a CRC patient if death occurred due to other causes:  Stage I: 23345  Stage II:21036  Stage III:26122  Stage IV:60707 |
| Complication costs, USD | Colon perforation: 15190  Serosal burn: 9812  Colonoscopy with bleeding: 1411  Colonoscopy severe bleeding (requiring transfusions): 8175 |
| Complication rates | Colon perforation rate: 0.07%  Serosal burn rate: 0.03%  Bleeding rate: 0.11%  Death after perforation rate: 5.2%  Death after severe bleeding (requiring transfusions) rate: 0.52% |
