# Supplementary material for: Optimal timing of a colonoscopy screening schedule depends on adenoma detection, adenoma risk, adherence to screening and the screening objective: A microsimulation study
Source: PLoS One. 2024 May 24;19(5):e0304374. doi: 10.1371/journal.pone.0304374 (PMC11125540; doi:10.1371/journal.pone.0304374)
Supplement: S3 Table — (DOCX) [file pone.0304374.s007.docx]

Additional file 3: Table S3:

**Input parameters for Mixed Integer Distributed Ant Colonoscopy Optimization (MIDACO)**

| Parameter | Number of colonoscopies | Minimum age (years) | Maximum age (years) | Time since last colonoscopy (years) | Compliance to screening colonoscopy (baseline) |
| --- | --- | --- | --- | --- | --- |
| Value range | 1..4 | 20 | 90 | 5..10 | 100% |
