# Supplementary material for: Optimal timing of a colonoscopy screening schedule depends on adenoma detection, adenoma risk, adherence to screening and the screening objective: A microsimulation study
Source: PLoS One. 2024 May 24;19(5):e0304374. doi: 10.1371/journal.pone.0304374 (PMC11125540; doi:10.1371/journal.pone.0304374)
Supplement: Attachment — Submitted filename: Point-by-point-response-to-reviewers-comments_26.03.2024.docx [file pone.0304374.s008.docx]

Dear Dr. Chih-Wei Tseng

We would like to thank you and the editors for the careful evaluation of our work. We modified our manuscript according to the suggestions of the reviewers. We are now able to submit what we feel is a much improved version of our work. We hope the text will now be suitable for publication in PLoS One. All changes are highlighted in the text and all our responses detailed in our point-by-point response letter.

V. Zaika, J. Poleszczuk and B. Misselwitz on behalf of the co-authors

Journal Requirements: When submitting your revision, we need you to address these additional requirements.

1. Please ensure that your manuscript meets PLOS ONE's style requirements, including those for file naming. The PLOS ONE style templates can be found at 
<https://journals.plos.org/plosone/s/file?id=wjVg/PLOSOne_formatting_sample_main_body.pdf> and 
<https://journals.plos.org/plosone/s/file?id=ba62/PLOSOne_formatting_sample_title_authors_affiliations.pdf>

*Response: this has been addressed.*

2. Thank you for stating the following financial disclosure: 
"Swiss Cancer League grant No. KFS-5164-08-2020 Benjamin Misselwitz."

Please state what role the funders took in the study.  If the funders had no role, please state: ""The funders had no role in study design, data collection and analysis, decision to publish, or preparation of the manuscript."" 
If this statement is not correct you must amend it as needed. 
Please include this amended Role of Funder statement in your cover letter; we will change the online submission form on your behalf.

*Response: this has been addressed.*

The [PLOS Data policy](http://www.plosone.org/static/policies.action#sharing) requires authors to make all data underlying the findings described in their manuscript fully available without restriction, with rare exception (please refer to the Data Availability Statement in the manuscript PDF file). The data should be provided as part of the manuscript or its supporting information, or deposited to a public repository. For example, in addition to summary statistics, the data points behind means, medians and variance measures should be available. If there are restrictions on publicly sharing data—e.g. participant privacy or use of data from a third party—those must be specified.

 Reviewer #1: No

Reviewer #2: No

*Response: We respectfully disagree. The code is fully available, and a link has been provided within the manuscript. The code can be used to reproduce all results from our manuscript.*

5. Review Comments to the Author

Reviewer #1: The paper discusses the optimal offering of colonoscopy for the CRC eligible population. Authors have used a publicly available microsimulation model for their analysis. I have several comments that need to be addressed. Some of them are more critical for publication:

1- Importance of the study:
Authors mentioned several reasons that they have done the analysis. Some of them are very valid. There are other reasons that can be added: 1- colonoscopy backlog: In many countries (including US), although screening with routine COL is suggested, the capacity of colonoscopy is limited and patients may need to wait for a long time to get a COL. Your analysis showcase better use of COL, potentially removing any backlog barrier. 2- In some other countries, such as Thailand, the capacity of COL is much fewer than industrilized countries, and health policy makers need to use such analysis to determine the time and interval of screening. Authors can take a look to the following publication L Wongseree, Peeradon, et al. "Dynamics of colorectal cancer screening in low and middle-income countries: A modeling analysis from Thailand." Preventive Medicine 175 (2023): 107694.

*Response: We agree with the ideas of the reviewer and modified the second paragraph of the introduction with the suggested reference (1) (page 3, line 15).*

However, authors mentioned that CISNET only have done a few COL intervals and start-stop age. The reason that CISNET does that were not probably the resources. This is what USPSTF instructed them to do. USPSTF has its own criteria and reasoning behind when and why the screening should start from age 45 instead of 47. For instance, ease of interpretation of results and ease of use of the results by physicians and patients have taken into account. The implementation of the proposed optimal solution in national level is very complicated, thus hinder USPSTF, ACS, ACP, USMSTF to advocate for it.

*Response: We would like to thank the reviewer for these comments. The reasoning for the CISNET group not to test yearly intervals is now better explained (page 3, line 33).*

2- Method:
Authors mentioned that they used CMOST model. It is expected to show the validity of the model compared to CISNET and other models in the US. Also external validation of the model, compared to clinical trials need to be shown. Here is an example of a model with validation points:
Vahdat, Vahab, et al. "Calibration and validation of the colorectal cancer and adenoma incidence and mortality (CRC-AIM) microsimulation model using deep neural networks." Medical Decision Making 43.6 (2023): 719-736.

*Response: We agree with the reviewer, that validation is crucial for any modelling study, and we read with great interest the recent study by Vahdat et. al. (2) (now quoted as new reference 44). However, we would like to respectfully note that CMOST validation had indeed been performed in the Paper by Prakash et al. (3), following a highly similar approach as used by Vahdat et al. (2). In brief, we performed a careful comparison of representative CMOST results with outcomes of CISNET microsimulation models (Figure 4 of the study by Prakash et al.). In addition, external validation had been performed, using data from the study by Schoen et al. (4), (compare Table 3, Effects of rectosigmoidoscopy screening for CRC prevention combined with 11.9-year follow-up from the study by Prakash et al.). Our results show that predictions by CMOST are in line with predictions of the CISNET model as well as clinical data.*

*However, we agree with the reviewer that this needs to be better explained in our manuscript. We now provide this relevant information in our manuscript (page 5, line 13). Further, benchmarks and results of our model calibrations are now provided (compare new Supplementary Table 1).*

Authors mentioned that "Due to the stochastic nature of the model, we performed 10 independent simulations for each set of evaluated model parameters". All simulation models are stochastic, but CISNET and others do not run the same model twice. I assume this is because the model is not using common random numbers (CRN). It is interesting for me to see that the model outcome fluctuate even with 20 million individuals (CISNET run their models with 2 million, as I recall).

*Response: We appreciate the insightful comments of this reviewer. Our model indeed does not use common random numbers. We rather use a set of 10 seed numbers as input parameters for the Mercenne-Twister uniform pseudo-random number generator.*

*We agree with the reviewer that some numbers in our results fluctuate, but not for simple outcomes of CMOST: the standard deviation is indeed quite small for incidence or mortality reduction due to colonoscopy (compare confidence intervals in Table 1). However, our numbers fluctuate for the dependencies of these results on model assumptions (e.g., Figures 1B, 2B, 3B), which represent complex calculations. One needs to remember that during optimization checking any new set of parameters requires stochastic simulation – this is another source of noise as there is non-zero chance that comparison between two sets of parameters will be different depending on the random seed value. To address this error, we consistently provide confidence intervals or other error measures. We now acknowledge and explain this fluctuation better in the new version of the manuscript (page 5, line 5).*

The use of MIDACO is very vague to me. Authors are encouraged to describe how the simulation and optimization models are connected. Are you using the simulation outcomes as inputs to optimization model? Or the optimization model is imbedded into simulation model and change the inputs of simulation model, based on previous findings?
Authors mentioned: "The optimization problem supplied to MIDACO was a standard nonlinear minimization (minimize incidence/ mortality/ life years lost/ total costs)". My first impression was that the optimization model is handling all these objectives simultaneously, which then the normalization and weights of these functions need to be clarified. If these objectives are handled separately, then the balanced solutions need to be further described.
MIDACO seems to use PSO meta-heuristic. None of the metaheuristic algorithms may guarantee the optimal solution. How does authors deal with that?

*Response: We want to apologise for the vague description of the optimization method. We have thoroughly reworked the section (page 5, lines 14-24) We are aware that none of the metaheuristic algorithms may guarantee optimal solution and that is exactly why in Figs. 1 – 3 we plot the landscape of the optimization problem to see if the resulting set of optimal parameter falls within the compact areas.*

Model inputs: the inputs to the model need to be clearly described and added as a table. These parameters include the costs, the adherence, adenoma detection rate, complication rates, and more.

*Response: We agree with the reviewer, the model inputs are now summarized in the new Supplementary Table 1.*

3-Results:
Frankly, the results are not clearly described. I suggest working on clarity of results.
Fig 1.B: While I see a meaningful pattern of changes in ADR and risk in age of screening, the same pattern does not exist for adherence. My interpretation from Figue1 B is that no matter of adherence, if you want to minimize the LYG or incidence it will be always the same age. This is in contrast to the literature. If this is the case, authors need to clearly explain their thoughts around it.

*Response: We agree with the reviewer, the results of Figure 1B should be described clearer and we are grateful to the reviewer for pointing this out. The optimal year for a single colonoscopy remains the same for individuals with high adherence as well as low adherence. This is due to the fact that for a screening program with a single colonoscopy (as assumed in Figure 1), a lower adherence simply results in non-participation of a fraction of the population in the program. This is now much better explained in the results explaining the findings in Figure 1B (page 7, line 20).* *We also show that for multiple colonoscopies adherence significantly impacts the optimal age of screening (see Fig 2B and Fig 3B). To increase clarity, we now provide reading examples in the legends to Figures 1B, 2B and 3B.*

4- Discussion:
I suggest authors to find one number for each screening interval and stick to it. Call out your number and let everyone knows what should be the exact time of screening if only two colonoscopy are offered.

*Response: We agree, our findings including the calculated optimal years for a 1-, 2- 3- and 4-colonoscopy schedule are now re-emphasized in the first paragraph of the discussion.*

We are not sue what USPSTF uses as their metric for choosing screening intervals. CISNET reports have LYG(benefit), cases averted(benefit), death averted (benefit), life-time colonoscopies (burden), complications (harms). CISNET are interested in benefit-to-burden ratios such as LYG per colonoscopies. It could be a good metric to optimize the model to. I refer authors the technical report of CISNET:
<https://www.ncbi.nlm.nih.gov/books/NBK570833/>

*Response: We agree with the reviewer. We added colonoscopies per LYG as well as colonoscopies per case prevented, as suggested by the reviewer to Table 1. We agree that LYG per colonoscopy would be an interesting metric for optimization, but in our opinion out of scope for the current study. This is now mentioned in the limitation sections (page 12, line 35).*

Reviewer #2: This is a paper which uses an existing microsimulation model, CMOST, to find optimal colonoscopy schedules for routine screening of asymptomatic individuals. It is a very interesting and useful topic, and one which has been generally underexplored. I commend the authors on tackling this, and providing a thoughtful analysis.
*Response: We are grateful to the reviewer for the general positive evaluation of our work.*

In general, I have concerns about the presentation of this paper, including how the modelling and statistical analysis are reported. The usefulness of this manuscript hinges on the reliability of the model, and understanding the precision and accuracy it can provide. In its current form, the manuscript does not convey to the reader any understanding of how accurate and/or precise the model is, how well it can replicate its targets, or any form of validation. I have noted below opportunities for the authors to add the required background; in the absence of these, the modelling is without foundation. There are some other minor points where the modelling is unclear; the use of the CHEERS checklist, or similar model evaluation, would help demonstrate the efficacy of their model and therefore the reliability of their results. There are also points where the statistical analysis and the results are reported in unclear ways; I have noted these below.

The authors should also acknowledge the work of Koffijberg et al, Using Metamodeling to Identify the Optimal Strategy for Colorectal Cancer Screening in Value in Health, 2020, which analyses optimal schedules for CRC via FIT screening.
*Response: We agree and added the reference to the paper (new reference 41, page 3, line 21).*

Page 3/Introduction:

Line 11: Country-level colonoscopy screening programs are not common, to my knowledge; I believe that it is mostly used ad-hoc or through insurance (as in the US) rather than as part of a national program. Sources 30-33 support this – 30 is about the British surveillance program, not screening (i.e. not for asymptomatic individuals), and 31-33 are for US guidelines, which is not a screening “program” per se. This needs to be clarified in your text, with specific country references.

*Response: We agree with the reviewer, there are not many country-based screening programs, and we now introduce CRC screening in more general terms without mentioning a program (page 3, lines 13-14). Previous reference 30 was removed.*

It is also worth noting that most country- or region-level screening programs for asymptomatic individuals are through FIT/FOBT (see e.g. Schreuders et al, Gut 2015). Many health-economic studies have shown that FIT is likely to be preferable vs colonoscopy for screening of an asymptomatic average-risk population (see e.g. Lew et al, International Journal of Cancer 2018, Zhong et al, Gastrointestinal Endoscopy 2019). However, there is no mention of FIT in your manuscript. Including this background, and the reasons colonoscopy would be used, would be very useful context for your manuscript.

*Response: We feel this is an important criticism and the first 3 paragraphs of the introduction have been rewritten and now introduce FIT as a highly cost-effective option; colonoscopy and FIT are subsequently discussed in a balanced way (page 3, line 6, 15) However, we feel that a discussion of the benefits of FIT vs. colonoscopy in even more detail would be out of scope for this paper.*

Lines 30-38 could be clarified into a clearer aim. Much of what is included is written as methods and results, and should be moved to those sections as appropriate.
*Response: We agree and shorted and rephrased the last paragraph of the introduction to make the aims of our study much clearer (page 3, line 37 until page 4, line 3).*

Page 4/Methods:
Line 4: It is unclear what re-engineered means – do you just mean you reimplemented the algorithms, or were they redesigned? If the algorithms have been changed, the differences from what has already been published should be described in detail. Otherwise please clarify the wording, as re-engineer implies a redesign of the model.

*Response: We agree with the reviewer and apologize for the unclear wording. The model has been re-implemented in C++ for much faster speed, while the logic and assumptions of the model have not been changed. This is now written much clearer in the new version of the manuscript (page 4. line 8).*

Line 6: As this includes a new set of calibration targets, these targets should be included in the manuscript (potentially in the supplementary materials), as well as the model performance on these calibration targets. As the meaningfulness of your manuscript hinges on the reliability of this model, this is critical.

*Response: We agree and provide our calibration results including the calibration to match the SEER data as a new Supplementary Table S1.*

I also note that in reference 35, the targets of the CMOST model are included as table S1 but it is not clear whether the model is able to successfully reproduce these targets. This should be included in this manuscript.

*Response: We agree and provide our calibration results as a new Supplementary Table S1.*

The SEER data only covers the US population, and therefore is most relevant to that population. As other countries can have dramatically different colorectal cancer incidence, mortality, and prevalence rates, you should make it clear to the reader throughout the manuscript, including in the introduction, that your baseline modelling is for the US population, though other populations could be approximated by the sensitivity analyses.

*Response: We agree and modified the wording accordingly (in the abstract, and at 14 additional occasions, e.g. page 3, line 38, page 4, line 7, 32, page 7, line 8).*

Line 13: It is very strange that your model includes CRCs with no precursor states. In Prakash et al 2017, it is stated that “this direct cancer pathway represents cancers with precursors difficult or impossible to detect by colonoscopy”. However, you are allegedly simulating a patient’s actual cancer/adenoma state (as evidenced by the simulation of screening, and the associated detection rates) rather than their observable (and potentially incorrect) state. This explanation is therefore a bit unsatisfactory. It seems to me that you are using this transition to “make up for” discrepancies between the data and your model performance. Perhaps this has to do with the timestep? A further explanation of this would be useful, as in the current manuscript it may appear that you are asserting that CRC can form spontaneously with no precursor, which I don’t think is the intention.

*Response: We appreciate the insightful comments of this reviewer. We would like to emphasize that the natural history of colorectal cancer is not understood in detail since observational studies without polypectomy are ethically not possible. There seems to be a significant fraction of CRC which are difficult to prevent (see below), most likely since they do not follow the simple adenoma – carcinoma path with adenomatous precursors detectable for a long time. One could imagine a number of non-mutually exclusive scenarios:*

- *There is evidence that in hereditary non-polyposis colorectal cancer at least some CRC directly derive from an intact epithelium and not an adenomatous precursor. In Lynch syndrome, these lesions can derive from mismatch-repair deficient crypt foci (MMR-DCF) which grow under an intact surface and are related to mutations in the CTNNB1 (5) or TP53 genes (6). Admittedly, the number of CRC with gate-keeper CTTNNB1 mutations is low (3%) (7) , however, a proof of principle of carcinoma without adenoma precursors has been established.*
- *Clinical experience suggests that some adenomas, especially in the right colon are exceedingly difficult to detect. Tandem colonoscopy (or CT-colonoscopy) is considered the gold standard for adenoma detection (8). However, this is clearly an imperfect gold standard for truly hard-to-find adenomas (flat inconspicuous lesions). Autopsy studies have been done, but to the best of our knowledge results have not been related to recent previous screening colonoscopies. Therefore, published adenoma detection rates might well underestimate the number of missed adenomas in the right colon.*
- *Very fast-growing adenomas with a dwell time <3 years (i.e. before the first surveillance colonoscopy after an adenomatous lesion) would have the same effect in our study as the “direct cancer” path (differences would only become obvious with very short screening/ surveillance intervals).*

*In our opinion there are not sufficient clinical (and pathological) data to distinguish between these three possibilities.*

*The assumption of the reviewer, that the “direct cancer path” had been introduced to adjust for discrepancies between our modelling results and clinical observations is correct. In our model the efficacy of CRC prevention by rectosigmoidoscopy in the right colon (due to diagnostic colonoscopy after a positive rectosigmoidoscopy) would be predicted to be much higher than observed in Atkin et al. (or similar studies) (9), and the “direct cancer path” was introduced and calibrated to “make up” for that difference (3). We don’t think that these discrepancies are due to the “time step” of three months in our model.*

*Generally speaking, for a simple microsimulation model with a uniform adenoma dwell time of 8-20 years of well-behaved, easily detectable lesions, CRC incidence reduction after colonoscopy would be unrealistically high. All microsimulation models have to adjust for this fact, chosing e.g. 1 (or more) of the above-mentioned options. None of these adjustments can be justified by hard data regarding the natural history of CRC (which is mostly unknown). Predictions of CMOST regarding CRC protection by screening measures are similar compared to CMOST (compare our detailed comparison in Prakash et al. (3)).*

*The direct cancer path and the reasoning explained above is now briefly summarised in the methods section (page 4, line 22).*

Line 18: Specify US guidelines.
*Response: We agree and specified the guidelines (page 4, line 29).*

Line 22: Is the three-month timestep a change from previous versions of CMOST? If so please make this explicit.
*Response: the 3-months time step has been used in CMOST 1 and CMOST 3 (no change). We now make this clear in the manuscript (page 4, line 10).*

Lines 29-32: Were these 10 simulations on the same parameter set? If so, it is unclear to me what the benefit of running these simulations, or of running the test afterwards is, vs combining the simulation outputs to increase the statistical power of your simulation (in these contexts, 20 million simulated individuals may not in fact be particularly “large”). Are these ten simulations the methods by which you generate the standard deviations included below? If so I don’t think these are meaningful standard deviations, as they are driven entirely by your choice of simulation size rather than anything inherent to the uncertainty of your model or the size of any population of interest.

*Response: We agree with the reviewer, the standard deviations and error bars in the Figures are derived from “technical replicas” with the same parameter set (but different seed values for the stochastic calculations). This has been done to illustrate the precision of our analysis with full transparency. Please keep in mind that CMOST is a complex microsimulation model and a population size >20 million in a single run would require much larger computational power than the cluster available to us. For complex outcomes such as the dependency of the optimal year on model assumptions (e.g., Figures 1-3B) unrealistically high numbers of individuals would be required to completely eliminate the uncertainty.*

*The impact of the uncertainty of model assumptions has been extensively investigated in our sensitivity analysis.*

*We now refer to the error bars as “precision of our analysis” in the Figure legends and provide some explanatory text in the method (page 19, line 11).*

Line 35-36: For each value required by the solver, was the model re-run, or was this a metamodel-type analysis like that in Koffijberg et al, Value in Health 2020?
*Response: For each input value required by the solver the whole model was rerun, except for the cases in which solver required inputs that were already calculated – we used local storage to recall already calculated outputs. This is now clarified in the methods section (page 5, line 17).*

Page 5

Line 14: What economic perspective was used? What costs are included?

*Response: we agree, costs from a healthcare payer’s perspective are used. This is now clearly stated in the manuscript (page 5, line 36).*

Line 28: This should be rewritten to make it clear that there is no fixed “current practice” for discounting, with various discount rates used based on setting and context. In particular the cost-effectiveness of screening programmes can be highly dependent on the choice of discount rate. A specific reference should be cited.
*Response: Thank you for the input, the respective text was rephrased and a supporting reference for the 3% discount rate has been provided (10).*

Line 36: Each parameter was changed separately, correct? If so this should be specified as a one-way sensitivity analysis.

*Response: We agree and rephrased the text accordingly (page 6, line 20).*

Line 36: Is the baseline adherence 100%?
*Response: We are grateful for the reviewer for pointing this out. Baseline assumptions are now provided in our new Supplementary Table S2. Baseline adherence to CRC screening is 100%, which is now also clearly stated in the text (page 6, line 25).*

Page 6

Lines 10-11: Based on my understanding, both the proportion and the relative risk of this “high-risk” group is entirely hypothetical and not based on actual groups within the population – is this correct? This should be made clearer, though the analysis is still very valuable.
*Response: We agree, our test is purely hypothetical. Is now phrased even clearer in the new version of the manuscript (page 6, line 35 and page 6, line 39).*

Page 8

Table 1:
This should be referenced in the text earlier.
*Response: We agree, Table 1 is now also referenced timely for the results of 1, 2, 3 and 4 screening colonoscopies (page 7, line 16).*

The asterisks do not seem to be referencing anything.
*Response: Thank you, the asterisks have been re-added to Table 1.*

“Standard screening” has not been defined in the text, as far as I can tell.

*Response: A standard screening is mentioned in the methods section. We now make it clearer by adding the hyphens (“”) also to page 6, line 17. The definition (years 50, 60, 70 years) is now one more time repeated in the results (page 8, line 15).*

What are these standard deviations based on?

*Response: We agree, this needs to be defined. The standard deviation indicates the precision of our calculations, based on 10 repetitions and this is now indicated in the legend to Table 1.*

What are these % changes vs? No screening? If so, the change in life-years gained is very strange. It is also not clear what the number in brackets for LYG is.

*Response: We are grateful to the reviewer for pointing this out. % changes refer to changes in life years lost. The numbers in brackets are life years gained. This is now better explained in the new header and legend of Table 1.*

The mortality reduction from a single colonoscopy also seems very high. Some reference to existing literature/validation of this rate would be useful context.

*Response: Up to 67% mortality reduction for the next 10 years after a single colonoscopy has been observed, which would be in line with a 40.6% life time mortality reduction for our optimized 1-colonoscopy screening schedule (11). In any case, these data indicate that a single colonoscopy has a disproportionally large benefit with the second (as well as the third and fourth) colonoscopy* *only provides incremental additional benefit. However, we respectfully point out that the focus of our paper is the optimal timing of colonoscopy screening interventions. Extensive discussion (or validation) of (arbitrary) individual absolute numbers would disrupt the flow of the paper, making it much less comprehensible. After careful consideration, the manuscript was not changed.*

Page 9

Discussion:
It should be noted that the “standard screening” scenario in Table 1 does in fact provide a good balance between the outcomes, based on the numbers you present; a partial endorsement of the current recommendations with scope to improve in line with your other findings.

*Response: We agree and modified the first paragraph of the discussion accordingly (page 11, line 2).*

Line 7: The “high risk”/”low risk” designators do not make sense in the context they are presented, where the “high risk” group can have lower cancer rates than the low-risk group in

Figure 5. I suggest you revise the wording around this analysis; perhaps just designate it as a “subgroup with a different risk”. The low risk also seems to be “average” or “general population” risk. This needs significant clarification.

*Response: We agree and modified the text accordingly (page 10, line 25-29).*

Line 26: It is strange to assert that LYG are “less intuitive” – you would be better off expanding on why they are less practical to measure.

*Response: We agree, wording needed improving. We mean that in a clinical study, life years gained cannot be directly measured since it is impossible to determine how long an individual patient would have lived without CRC-related death. Further, in our clinical practice, discussions with patients typically focus on CRC prevention and/ or mortality prevention but never on LYG. However, in the absence of data on patient preferences this is now phrased more carefully (page 11, line 13-19).*

A limitation of your study is that you have used life-years, rather than quality-adjusted life years, as a measure; this should be mentioned and the difference noted (especially when considering high-risk groups).
*Response: We agree and adjusted the limitations section (page 12, line 40).*

Page 10:

Line 13: Unclear what “high performance” means here.

*Response: We meant high computational speed. But this is likely beside the point and we removed this term (page 12, line 3).*

Line 22: This first sentence is incomplete.

*Response: We agree and modified accordingly (page 12, line 12).*

Line 29: Although this is a strong conclusion, “recommending early start for those with strong adherence” is a meaningless recommendation in practice – you can only know this retroactively! Suggest removing or recontextualising.

*Response: We agree, this is an important point. We still feel that the best use of this observations is informing the decision of a patient and his/ her health care provider. We now state the concern of the reviewer clearly in the discussion and provide some suggestions, how adherence could possibly be predicted (page 12, line 16-22)..*

Good suggestion of moving towards personalised recommendations; you could argue that your findings would be useful to inform ad-hoc recommendations for clinicians to inform people who are not sure if they should receive a colonoscopy or wait.
*Response: Thank you for this suggestion. We modified the text accordingly (page 12, line 23-27).*

Figure 1: Suggest moving B to supplementary materials, or finding a simpler way to convey this.

*Response: We agree, we reworked Figure 1B, making it less crowded and more comprehensible. The sensitivity analysis regarding total costs was moved to the Supplement. A reading example for the sensitivity analysis (which constitutes a core part of our paper) is now provided in the legend to Figures 1B, 2B and 3B (page 19, line 12-17; page 19, line 27-30, page 20 line 1-4).*

Figure 2: A) I do not understand the advantage of including the non-dominated solutions – the colours are sufficiently clear. “Effectiveness” is misspelled. Again B is not sufficiently clear for inclusion in the main text, in my opinion.

*Response: We agree, the spelling of “effectiveness was corrected, and we reworked Figure 2B to make it less crowded and more comprehensible, with a reading example (page 19, line 25-27).*

*The Pareto solutions provide additional relevant information, informing about solutions where no single optimal schedule can be identified for one outcome, without compromising on another. We feel, that plotting the PARETO solutions illustrate the fact that in the presence of multiple objectives, each solution is a compromise. We now briefly explain this reasoning in the results (page 5, line 21, remind the reader that PARETO solutions are compromises repeatedly in the results).*

Figure 3: Presenting this as a 3D figure has no advantage over presenting it as three 2D figures, side by side, and makes it more difficult to read. It is not possible for a reader to visually interpret the line indicating the optimum on the z-axis. Again, omitting the non-dominated solutions will improve readability, and figure B should be moved to supplementary material.

*Response: We reworked Figure 3B, making it less crowded, more comprehensible and provide a reading example. We now also refer to the Pareto solutions in the text (page 7, line 17). We respectfully disagree regarding Figure 3A. We agree that the exact numbers of the plots (e.g. of the 95% effectiveness zone) are more difficult to read in a 3D plot. However, for our study it is more important to illustrate the concept that the optimum screening schedule for this scenario needs to be searched for in a 3D space, and for this a 3D illustration is suited best.*

Figure 4: Unclear to me how this is an “ICER analysis”; the outcome listed is the discounted LYG? The ICER is not shown on this figure, as far as I can tell.
*Response: Thank you, we agree. There is no ICER on this graph and the caption to figure 4 has been modified (page 20, line 5-8). .*

Figure 5: As noted in the text, “high” and “low” risk are very misleading when the low-risk group can have risk ratios less than 1.

*Response: We agree and modified the figure legend and the results accordingly (page 20, line 9-14).*

# References

1.	Wongseree P, Hasgul Z, Leerapan B, Iramaneerat C, Phisalprapa P, Jalali MS. Dynamics of colorectal cancer screening in low and middle-income countries: A modeling analysis from Thailand. Preventive Medicine. 2023;175:107694.

2.	Vahdat V, Alagoz O, Chen JV, Saoud L, Borah BJ, Limburg PJ. Calibration and Validation of the Colorectal Cancer and Adenoma Incidence and Mortality (CRC-AIM) Microsimulation Model Using Deep Neural Networks. Med Decis Making. 2023;43(6):719-36.

3.	Meher K. Prakash BM. CMOST: an open-source framework for the microsimulation of colorectal cancer screening strategies. 2017;17.

4.	Schoen RE, Pinsky PF, Weissfeld JL, Yokochi LA, Church T, Laiyemo AO, et al. Colorectal-Cancer Incidence and Mortality with Screening Flexible Sigmoidoscopy. N Engl J Med. 2012;366(25):2345-57.

5.	Lepore Signorile M, Disciglio V, Di Carlo G, Pisani A, Simone C, Ingravallo G. From Genetics to Histomolecular Characterization: An Insight into Colorectal Carcinogenesis in Lynch Syndrome. International Journal of Molecular Sciences [Internet]. 2021; 22(13).

6.	Ahadova A, Gallon R, Gebert J, Ballhausen A, Endris V, Kirchner M, et al. Three molecular pathways model colorectal carcinogenesis in Lynch syndrome. Intl Journal of Cancer. 2018;143(1):139-50.

7.	Arnold A, Tronser M, Sers C, Ahadova A, Endris V, Mamlouk S, et al. The majority of β-catenin mutations in colorectal cancer is homozygous. BMC Cancer. 2020;20(1):1038.

8.	Zhao S, Wang S, Pan P, Xia T, Chang X, Yang X, et al. Magnitude, Risk Factors, and Factors Associated With Adenoma Miss Rate of Tandem Colonoscopy: A Systematic Review and Meta-analysis. Gastroenterology. 2019;156(6):1661-74.e11.

9.	Atkin WS, Edwards R, Kralj-Hans I, Wooldrage K, Hart AR, Northover JM, et al. Once-only flexible sigmoidoscopy screening in prevention of colorectal cancer: a multicentre randomised controlled trial. The Lancet. 2010;375(9726):1624-33.

10.	Li Q, National Bureau of Economic R, Pizer WA. Discounting for Public Cost-Benefit Analysis: National Bureau of Economic Research; 2018.

11.	Effectiveness of screening colonoscopy in reducing the risk of death from right and left colon cancer: a large community-based study. Gut. 2018;67:291-8.
