# Supplementary material for: Optimal timing of a colonoscopy screening schedule depends on adenoma detection, adenoma risk, adherence to screening and the screening objective: A microsimulation study
Source: PLoS One. 2024 May 24;19(5):e0304374. doi: 10.1371/journal.pone.0304374 (PMC11125540; doi:10.1371/journal.pone.0304374)
Supplement: Attachment — Submitted filename: Point-by-point-response-to-reviewers-comments_23.04.2024.docx [file pone.0304374.s009.docx]

Dear Dr. Chih-Wei Tseng

We would like to thank you and the editors for the careful evaluation of our work. We modified our manuscript according to the suggestions of the reviewers. We are now able to submit what we feel is a much improved version of our work. We hope the text will now be suitable for publication in PLoS One. All changes are highlighted in the text and all our responses detailed in our point-by-point response letter.

V. Zaika, J. Poleszczuk and B. Misselwitz on behalf of the co-authors

Journal Requirements:

Please review your reference list to ensure that it is complete and correct. If you have cited papers that have been retracted, please include the rationale for doing so in the manuscript text, or remove these references and replace them with relevant current references. Any changes to the reference list should be mentioned in the rebuttal letter that accompanies your revised manuscript. If you need to cite a retracted article, indicate the article’s retracted status in the References list and also include a citation and full reference for the retraction notice.

*Response: The references have not been modified.*

**Additional Editor Comments:**

Please ensure that your manuscript meets PLOS ONE's style requirements, including those for file naming. For example, the simple summary section is not necessary

*Response: We agree, we double checked the style requirements. The summary section has now been deleted (page 2).*

**Comments to the Author**

6. Review Comments to the Author

Reviewer #2: Most of my concerns have been well-addressed, especially with the inclusion of Supplementary Table 1. As this table shows that the calibration of the model is good, but perhaps not perfect, I would appreciate if the authors could mention the quality of the calibration in the results or discussion. The authors should be able to quantify the discrepencies between the targets and the model, and provide commentary on how accurate the model is and how useful the findings based on it are (I personally think it would be very reliable).

*Response: We agree and commented on the quality of the fit and possible consequences for the reliability of our mode (pages 4-5, lines 33-5).*

Table 1 - The "Life-years gained" outcome still needs to be rewritten if the percentage is your main outcome. Unless I am misunderstanding, you mean "Reduction in life-years lost to CRC death" - correct? The distinction is very important to make the comparator clear, as otherwise it appears as "Number of life-years in the total US population".

*Response: We agree and modified the text accordingly. Life years gained is now appropriately defined and we also provide the definition in the headings of Table 1 (Table 1, page 10, line 4).*

These small changes would improve the manuscript. Otherwise, I strongly recommend it for publication as a useful and fascinating addition to the literature on colonoscopy screening.

*Response: We would like to thank this reviewer for his efforts during this very constructive reviewing process and the general appreciation of our work.*
